# Blue Zone Region Older Adults Mitigate ACEs and Other Chronic Stress Exposures

**DOI:** 10.1093/geroni/igaa057.3357

**Published:** 2020-12-16

**Authors:** Fatimah Alramadhan, Manisa Bhuyan, Rhonda Spencer

**Affiliations:** 1 Loma Linda University, Loma Linda, California, United States; 2 Texas State Health Department, Huntington Beach, California, United States

## Abstract

Adverse Childhood Experiences (ACEs) has been linked to many negative health outcomes, including shortened lifespan. ACEs are prevalent in the US, where it is estimated that one in ten have experienced at least three ACEs. Knowing the adverse ACEs impact, it is important to understand mitigating factors, leading to healthier and longer life. Loma Linda, CA has been identified as a longevity hotspot region (LHR) and may provide insight into reducing impact of chronic stressors. Our purpose to interview LHR older adults regarding their childhood experiences to inform chronic disease prevention framework. We conducted a qualitative study with LHR community members. Childhood exposures and practices assessed using semi-structured key informant interviews (KIs), with questions informed by ACE International Questionnaire and supplemented with lifestyle and resiliency factor (RFs) questions. Data were audio recorded and transcribed. Integrative grounded theory methods guided coding and theming. Participants included 26 community members (14 aged 100 or older and 12 aged between 90 and 99) who reported numerous chronic stressors including ACEs and environmental exposures (i.e. household economic depression). Correlation analysis revealed overlapping of negative experiences. Several domains of protective themes (i.e. guiding presence, spirituality)—were identified in childhood, with potential to dampen chronic systemic inflammation—and many themes maintained across their life. Our findings show that even among older adults who are 90 or older there is a high prevalence of ACEs and stressors; however, many lifestyle protective factors were practiced by them, which may offset damage of stressors and positively influence health and longevity.

